# Gender-disease interaction on brain cerebral metabolism in cancer patients with depressive symptoms

**DOI:** 10.1186/s12888-018-2002-6

**Published:** 2019-01-08

**Authors:** Zhijun Yao, Lei Fang, Yue Yu, Zhe Zhang, Weihao Zheng, Zhihao Li, Yuan Li, Yu Zhao, Tao Hu, Zicheng Zhang, Bin Hu

**Affiliations:** 1 Gansu Provincal Key Laboratory of Wearable Computing, Lanzhou University, Lanzhou, Gansu Province 730000 People’s Republic of China; 2 Center of Excellence in Brain Science and Intelligence Technology Chinese Academy of Sciences(CEBSIT), Shanghai Municipality, 200031 People’s Republic of China; 3 PET/CT Center, Affiliated Lanzhou General Hospital of Lanzhou Military Area Command, 333 South Binhe Road, Lanzhou, 730050 Gansu Province People’s Republic of China

**Keywords:** Depression, Gender, Cancer, Metabolic abnormalities, 18F-FDG PET

## Abstract

**Background:**

Cancer patients are accompanied with high morbidity of depression, and gender effects are known to inhabit in the depressive episodes. This study aimed to explore the gender effects in cancer patients, and the relationship between gender-cancer factors and the depression symptoms.

**Methods:**

The 18F-FDG PET scans of 49 cancer patients and 48 normal controls were included. We used voxel-wise analysis to explore the effects of cancer factor and gender factor in cerebral glucose metabolism. Beck Depression Inventory was utilized to quantify the depression symptoms in cancer patients.

**Results:**

Our results showed significant cancer main effects primarily in superior frontal gyrus and parietal gyrus; and significant gender main effects primarily in cerebellum posterior lobe, inferior temporal gyrus. Significant gender-by-cancer interaction effects were also observed, which primarily located in superior frontal gyrus. We showed the metabolic intensities of the 5 aforementioned clusters were related to the mental stress of depressive emotion.

**Conclusions:**

Our results suggested that males and females have different psychological endurance when facing cancer diagnosis or preventing depression. Furthermore, the cerebral abnormal metabolism might serve as a depressive indicator for cancer patients. The present findings provided supporting evidence for abnormal cerebral glucose metabolism affected by gender factor in cancer patients with mental stress of depressive emotion, and these brain regions should be concerned in clinic.

## Background

As a source of suffering, depression is often omitted by cancer patients [1]. Previous study indicated that acute stressful life events can lead to first onset of depression and recurrence of episodes of major depression [2]. Cancer diagnose, as an acute stressful life event, is considered as an important factor inducing psychological and emotional stress, and is beyond the coping mechanisms of patients which may result in major depressive disorder [3]. Depression can reduce the life quality of cancer patients and may negatively influence compliance with medical treatment [4–7]*.*

Studies of depression morbidity in cancer patients have shown significant differences between females and males in depression state [8–10]. Moreover, females showed worse anxiety and depression compared to males (even two or three times higher in some types of cancer) [11, 12]. Thus, for depressive cancer patients, gender effect was always one of the indicators. However, previous studies regarding gender differences in depression only used the behavioral/psychosocial analysis [13–15], abnormalities in metabolism affected by gender is not yet well examined in cancer patients with mental stress of depressive emotion.

A large number of therapeutic clinical trials have proposed using the metabolic indicator to assess therapeutic response rather than relying on conventional anatomical measures. Because the properties of positron decay permit accurate imaging of the distribution of positron-emitting radiopharmaceuticals, positron emission tomography (PET) assessment in 18F-fluordeoxy glucose (FDG) uptake is gaining acceptance as such a measure [4]. The wide array of positron-emitting radiopharmaceuticals has been used to characterize multiple physiologic and pathologic states, and the 18F-FDG PET was indicated to provide useful metabolic information for cancer patients [5].

18F-FDG PET and voxel-wise analytical method were used in this study to explore the interaction of gender and cancer factors in depressive cancer patients on glucose metabolic abnormalities. 18F-FDG uptake, which would provide potential metabolic information, is a crucial indicator in brain studies and has been widely adopted as an important measurement which could reflect brain activities from the perspective of metabolism [7, 16]. Yao et al. analyzed the modular pattern reconfiguration of metabolic brain networks using the 18F-FDG PET in cancer patients [6]. Besides, Tashiro et al. investigated the influences of depressive states on the regional brain activity of cancer patients by the 18F-FDG PET images [7]. Based on the reported gender-related differences and metabolic abnormalities in depressive cancer patients, we hypothesized that metabolic intensity of the brain may be influenced by gender and cancer factors and may relate to the mental stress of depressive emotion. In present study, PET images were used to explore the main effects and interaction effects of gender factor and cancer factor, as well as the relationship between mental stress of depressive emotion and cerebral metabolism. The present study provided evidence on depression studies with the 18F-FDG PET brain images analyzed by the PET voxel-wise analytical method, and may support gender difference research in neuroimaging.

## Methods

### Subjects

In the present study, 49 cancer patients (35 males, 14 females, age 50.918 ± 9.338) and 48 normal controls (NCs) (30 males, 18 females, age 50.604 ± 7.721) were recruited. PET images were gathered between August 2014 and December 2015 and the privacy of participants was guaranteed. The inclusion criteria for cancer patients were as follows: (a) age at 18 or older at the time of diagnosis, (b) ability to tolerate 18F-fluorodeoxyglucose injection, (c) no cancer type which could be found only in males or females, (d) well-informed about his or her own condition, (e) knew about his or her disease within 1 year, (f) absence of clear focal brain lesions or mental diseases, (g) did not undergo chemotherapy, (h) no more than 2 years of cancer. The NCs should have no psychiatric disorder or mental disease, were all confirmed with no depression or depressive symptom by the doctors. We used Beck Depression Inventory (BDI)-II to assess the mental stress of depressive emotion in cancer patients [8]. All participants were given written informed consent at the time of enrollment for PET image scanning, according to the Declaration of Helsinki (1991).

### Sample description

The demographic and clinical measures between cancer patients and NCs were listed in Table 1. The types of cancer patients were as follows: lung cancer (*n* = 9, female = 3), bowel cancer (*n* = 7, female = 2), lymph cancer (*n* = 7, female = 4), gastric cancer (*n* = 6, female = 1), renal cancer (*n* = 2, female = 0), esophagus cancer (*n* = 2, female = 0), nasopharynx cancer (*n* = 2, female = 1), other types of cancer (*n* = 14, female = 3). The cancer patients included 28 minimal depressions (score 1–13, mean value: 6.50; SD: 3.45), 11 mild depressions (score 14–19, mean value: 15.55; SD: 1.63) and 10 moderate/severe depressions (score 20–63, mean value: 25.20; SD: 3.49). Chi-square test and two-sample T test was used, respectively, to examine the difference in gender and age between the two groups. No significant difference were found in age and gender (*p* > 0.05).

**Table 1:** Demographic characteristics, clinical measures in cancer patients and normal controls

	CAN		CON		*P*-value
	Male(35)	Female(14)	Male(30)	Female(18)	
Age(years)	50.29 ± 9.69	52.50 ± 8.53	50.31 ± 7.23	51.11 ± 8.58	0.350
BDI	11.75 ± 8.09	14.00 ± 8.38	–	–	0.399

*CAN* cancer, *CON* normal controls, *BDI* the Beck Depression Inventory. Mean and standard deviations (±) are given

### PET image acquisition and analysis

PET images of all participants were gathered using a Siemens Biograph TruePoint 64 PET/CT (Siemens Healthcare, Erlangen, Germany) in three-dimensional mode at Affiliated Lanzhou General Hospital of Lanzhou Military Area Command. The image collection of coregistered CT and PET images was performed together. Preparation rules of all participants were strictly followed during the acquisition process. In order to keep the blood glucose level within the scope of 3.9–6.1 mmol/L, all the participants were required to fast for at least 6 h. Seven minutes scan was acquired in 40–60 min after intravenous injection of 3.7 MBq/kg (maximum dose 370 MBq) of 18F-FDG. In this study, the CT data on the combined scanner were used for PET attenuation correction. The FDG-PET data were reconstructed with ordered subset expectation maximization iterative algorithm.

All the images were preprocessed using the Statistical Parametric Mapping software *(SPM8,* *http://www.fil.ion.ucl.ac.uk/spm/software**)* in MATLAB 7.14, and were normalized to the standard image data for further analysis. The spatial normalization with a 12-parameter affine transformation was used, followed by nonlinear iterative spatial transformation. PET voxel-wise analysis was used to analyze the cerebral metabolism, and the voxel size after normalization was 1 mm × 1 mm × 1 mm. Finally, the processed images were smoothed by using 8 mm FWHM Gaussian kernel.

### Statistical analysis

Demographic and clinical measures were tested by Chi-square test and two-sample t-test, respectively, in SPSS22. The whole brain metabolic analysis was performed using full factorial analysis model in SPM8. In this model, the PET images were entered into a voxel-by-voxel general linear model, with a 2 (disease: cancer vs. NCs) × 2 (gender: female vs. male) two-way analysis of variance (ANOVA), to explore the cancer main effect, the gender main effect and the gender-by-cancer interaction effect. The main effect analysis of each factor was performed in all levels of the other factor. We set the extent threshold of per cluster to 100 contiguous voxels to ensure the validity of the results. An alpha level of *p*-values < 0.05 corrected for family-wise error (FWE) were considered to be significant.

In order to explore the correlation between cerebral metabolism and depression state, we calculated the Pearson correlation coefficient voxel-by-voxel between the voxels restricted to significant clusters and BDI scores (a voxel-wise analysis) in the cancer group, and then counted the number of significant voxels that were related to the BDI scores. A false discovery rate (FDR) correction was performed at a p-value of 0.05 for multiple comparison correction. Locations of the significant brain regions were conducted with xjview Toolbox *(**http://www.alivelearn.net/xjview/**)* and BrainNet Viewer Toolbox [9].

## Results

### Effects of gender and cancer interaction on cerebral metabolism

**Fig. 1:**
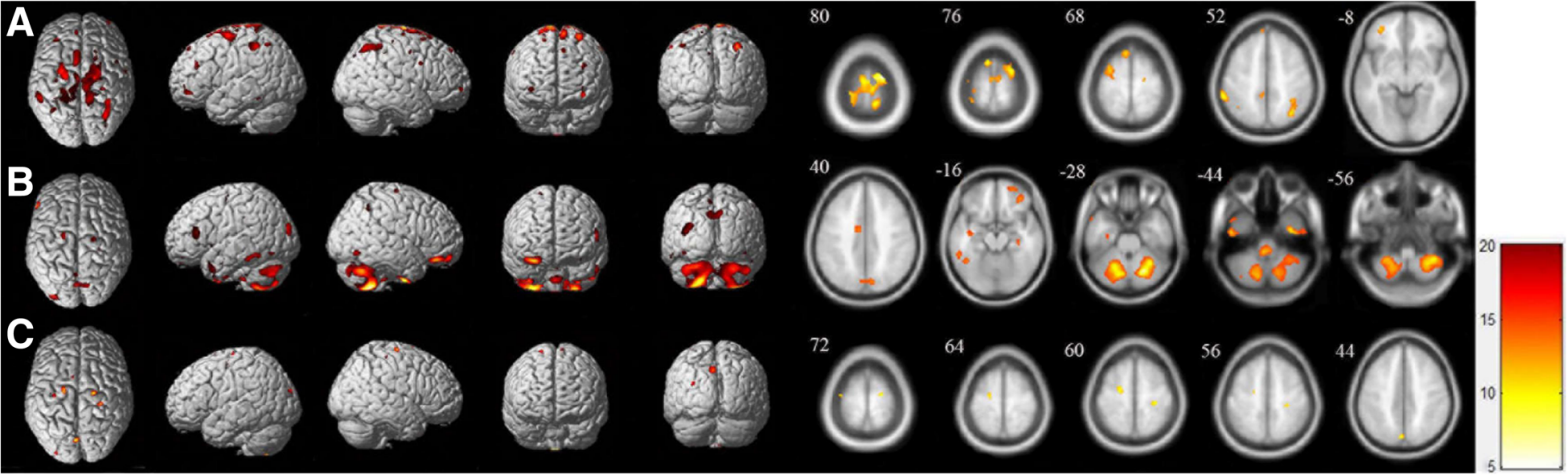
Brain mapping depicting the combined effects of cancer and gender in two-way ANOVA. **a** Significant cancer main effect clusters primarily located in frontal lobe, parietal lobe and supplementary motor area (P_FWE-correct_ < 0.05, 100 contiguous voxels per cluster). **b** Significant gender main effect clusters primarily located in cerebellum posterior lobe, temporal lobe, cuneus and cingulate cortex (P_FWE-correct_ < 0.05, 100 contiguous voxels per cluster). **c** Significant gender-by-cancer interaction effect clusters primarily located in frontal lobe, cuneus, and postcentral gyrus (P_FWE-correct_ < 0.05, 100 contiguous voxels per cluster)

As is shown in Tables 2, 8 clusters with significant cancer main effect were found by using two-way ANOVA, in which the largest cluster located in the left superior frontal gyrus (BA 6;MNI coordinates:x = − 21, y = − 12, z = 63). Other clusters were located in right superior parietal gyrus, left supplementary motor area, left middle part of orbitofrontal gyrus, left inferior parietal gyrus, left superior frontal gyrus: medial, and left median cingulate and paracingulate gyrus. The clusters with significant cancer main effect were shown in Fig. 1. We also found 7 clusters with significant gender main effect, in which the largest cluster located in bilateral cerebellum posterior lobe (right MNI coordinates: x = 22, y = − 68, z = − 30, left MNI coordinates: x = − 17, y = − 67, z = − 32), other clusters were located in left inferior temporal gyrus, right cuneus, left median cingulate and paracingulate gyrus, left postcentral gyrus, and right superior frontal gyrus. We also found 4 clusters with significant gender-by-cancer interaction effects, which mainly located in bilateral superior frontal gyrus, right postcentral gyrus, left precuneus, and left superior frontal gyrus(the largest cluster, BA 6; MNI coordinates: x = − 20, y = − 12, z = 61). The information of clusters were shown in Fig. 1 and Table 2.

**Table 2:** Results of the gender and cancer factors two-way ANOVA of the 18F-FDG PET brain image

Two-way ANOVA	Cluster size	Anatomical region	Hemisphere	BA	F-value	Peak coordinates(x,y,z)(mm)
Cancer main effect	2030	Frontal_sup	Left	6	13.2	−21 -12 63
	1551	Parietal_sup	Right	40	11.59	33 –63 52
	1066	Supp_motor_area	Left	6	12.46	−7 8 74
	374	Frontal_mid_orb	Left	–	10.55	−33 47 –8
	317	Frontal_mid	Right	40	11.59	33 –63 52
	143	Cingulum_mid	Left	5	8.95	−7 -39 51
	141	Parietal_inf	Left	40	8.7	38 –57 54
	138	Frontal_sup_medical	Left	8	9.33	−7 46 50
Gender main effect	21,928	Cerebellum posterior lobe	Right	–	23.41	22 –68 -30
	20,683	Cerebellum posterior lobe	Left	–	19.01	−17 -67 -32
	1195	Temporal_inf	Left	20	11.63	−44 -51 -14
	1100	Cuneus	Right	7	11.91	11 –76 42
	495	Cingulum_mid	Left	24	10.37	−10 -6 39
	378	Precentral	Left	6	10.54	−21 -11 62
	127	Frontal_sup	Right	6	9.53	20 –14 70
Interaction	356	Frontal_sup	Left	6	16.44	−20 -12 61
	305	Precuneus	Left	7	14.67	−4 -76 43
	233	Postcentral	Right	4	16.33	28 –29 59
	209	Frontal_sup	Right	6	17.40	19 –14 70

For each significant cluster, we reported the F-value (peak intensity) and MNI coordinates at the position of the maximum, the cluster size (k) and the corresponding Brodmann area (BA). Only clusters with k > 100 are shown

### Simple effects analysis of the main effect regions

**Fig. 2:**
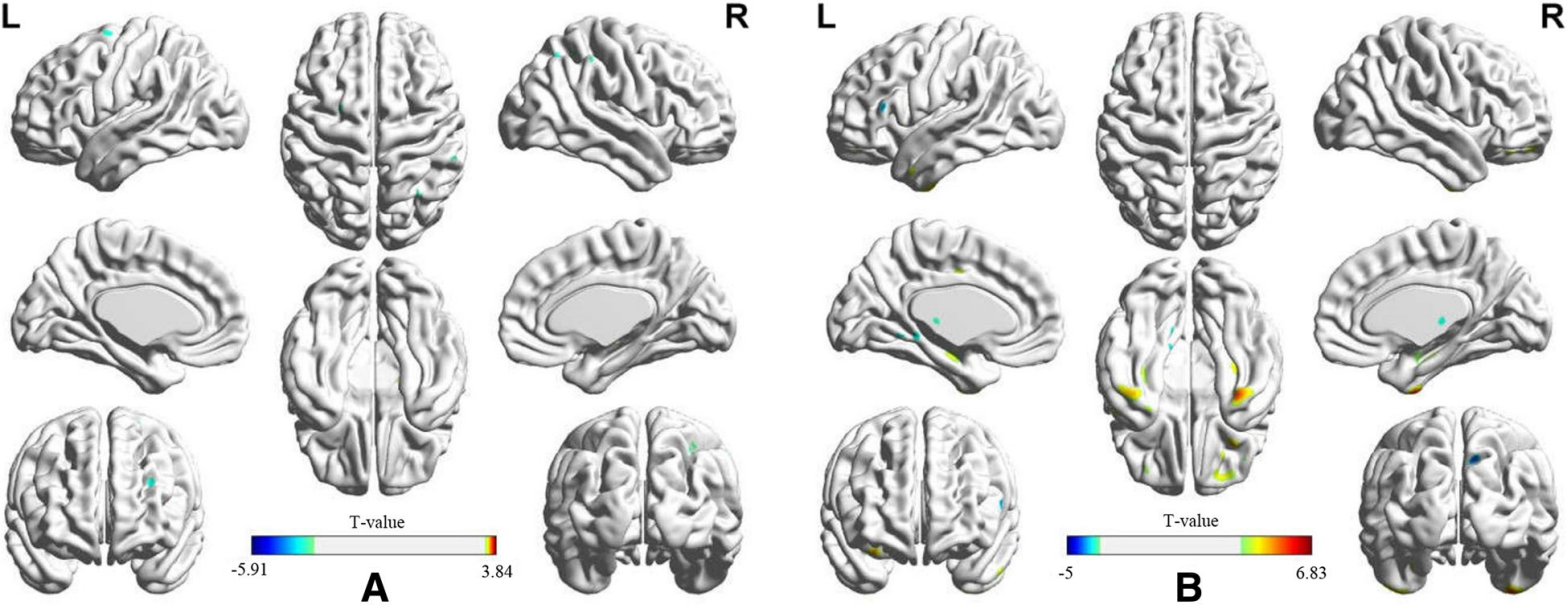
Clusters of post hoc test results. **a** Clusters related to cancer factor (positive: cancer > control, negative: cancer < control). **b** Clusters related to gender factor (positive: male > female, negative: male < female)

Results from post-hoc test showed significant decrease of metabolic intensity in superior frontal gyrus and postcentral of the cancer patients relative to controls. No significant hypermetabolic cluster was found in cancer patients. We found 5 clusters with significant decrease of metabolic intensity in female compared to male, which were located in right parahippocampal gyrus, right cingulate gyrus, left median cingulate and paracingulate gyrus, left anterior cingulate and paracingulate gyrus and right thalamus. Whereas, 8 clusters were found with significant increase of metabolic intensity in female compared to male that were located in right cuneus, bilateral inferior parietal lobules, right median cingulate and paracingulate gyrus, left postcentral, right precuneus, right supplementary motor area, and left superior occipital gyrus (see Table 3 and Fig. 2).

**Table 3:** Results of the post hoc test

Post hoc T	Cluster size	Anatomical region	Hemisphere	BA	T-value	Peak coordinates(x,y,z)(mm)
Cancer < Control	382	Superior frontal gyrus	Right	6	5.91	19 –11 81
	170	Postcentral	Right	7	5.11	11 –42 83
Male > Female	19,542	Parahippocampal gyrus	Right	–	4.43	33 –23 -20
	7232	Cingulum_ant	Left	32	4.06	−12 31 14
	4373	Cingulum_mid	Left	24	4.50	−10 -6 39
	3336	Cingulate gyrus	Right	24	3.58	18 4 41
	1920	Thalamus	Right	–	3.29	22 –21 13
Male < Female	6275	Cuneus	Right	7	4.84	11 –76 42
	1483	Parietal_inf	Right	40	3.60	56 –51 53
	1127	Cingulum_mid	Right	31	3.18	1 –32 43
	949	Parietal_inf	Left	7	3.32	−29 -56 43
	864	Precuneus	Right	7	2.91	6 –64 56
	856	Postcentral	Left	–	3.80	−30 -35 77
	353	Occipital_sup	Left	–	3.04	−12 -86 46
	338	Sup_motor_area	Right	6	2.93	4 –3 71

For each significant cluster, we reported the T-value (peak intensity) and MNI coordinates at the position of the maximum, the cluster size (k) and the corresponding Brodmann area (BA). Only clusters with k > 100 are shown

### Correlation between cerebral metabolism and mental stress of depressive emotion

**Fig. 3:**
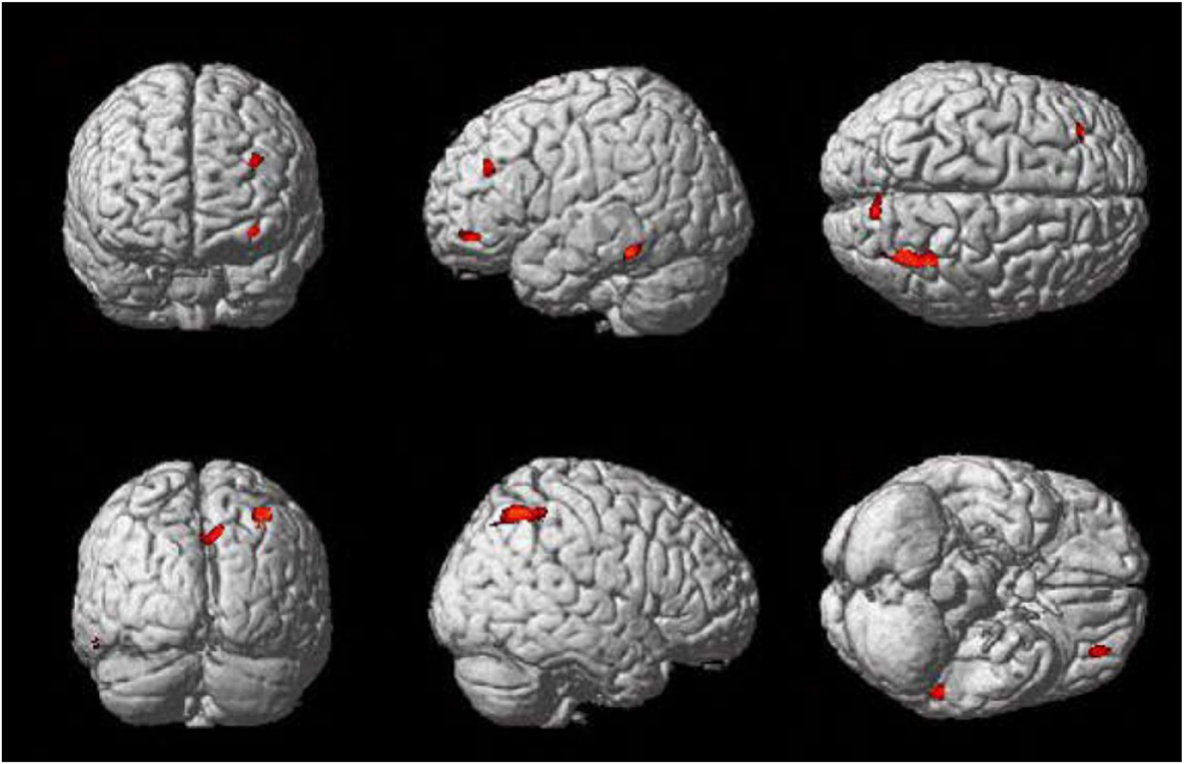
Size and location of the clusters whose metabolic intensity was significantly related to the BDI score (P_FDR-correct_ < 0.05, 100 contiguous voxels per cluster)

The correlation analysis showed significant correlation between BDI score and voxels within the clusters of cancer main effect, which mainly located in right superior parietal gyrus, middle orbitofrontal gyrus and left middle frontal gyrus (q < 0.05,FDR corrected). Voxels within the clusters of gender main effect that showed significant correlations with BDI score mainly located in right cuneus and left inferior temporal gyrus(q < 0.05, FDR corrected). The detailed information were shown in the Table 4 and Fig. 3.

**Table 4:** Size and location of voxels that showed relationship between metabolic intensity and BDI score

Two-way ANOVA	Correlative voxel	Anatomical region	Hemisphere	correlation coefficent	*P*-value	Peak coordinates(x,y,z)(mm)
Cancer main effect	1106	Parietal_sup	Right	−0.3174	0.0262	35 –46 49
Gender main effect	372	Cuneus	Right	−0.3289	0.0207	9 –76 46
Gender main effect	277	Temporal_inf	Left	−0.2965	0.0386	−55 -42 -15
Cancer main effect	224	Frontal_mid_orb	Left	−0.3446	0.0153	−34 44 –11
Cancer main effect	210	Frontal_mid	Left	−0.3163	0.0269	−32 38 30

## Discussion

In the present study, we explored the interaction of gender and cancer factors on cerebral glucose metabolism in cancer patients and normal controls. We found several clusters whose metabolic intensity was affected by gender and cancer factors. Additionally, correlation analysis showed the relationship between these affected clusters and the mental stress of depressive emotion. Our results indicated that gender and cancer factors could induce changes in brain metabolism and these metabolic changes are associated with the mental stress of depressive emotion.

### Brain regions affected by the gender and cancer factors

Details of the regions affected by gender and cancer factors are shown in Table 2 and Fig. 1. Significant cancer main effects have been found primarily in the frontal lobe, parietal lobe and the cingulate gyrus. Post hoc analysis showed the hypermetabolic regions primarily located in the frontal gyrus and postcentral in cancer patients compared with NCs. Our results were consistent with the previous studies showing the metabolic abnormalities in cingulate gyrus, orbitofrontal gyrus and dorsolateral prefrontal gyrus in cancer patients [7, 17]. The abnormal metabolism in cingulate gyrus and dorsolateral prefrontal gyrus in cancer patients was consistent with the previous findings that cingulate gyrus and dorsolateral prefrontal gyrus were more objective and validate biomarkers in emotional processing and depression diagnosis [10]. Besides, these regions might be associated with the depth of depression [7]. The abnormal metabolic regions in the frontal lobe may help to explain the dysfunction of the brain activities after the diagnose of cancer [11]. Our results suggested that the cancer factor may result in the abnormal metabolic intensity in these regions which might be associated with the mental stress of depressive emotion.

As is shown in Table 2 and Fig. 1, significant gender main effects were found primarily in the bilateral cerebellum posterior lobe, the temporal lobe, cuneus and frontal lobe, with the hypermetabolic regions primarily located in the parahippocampal gyrus and the cingulate gyrus, and the hypometabolic regions primarily located in the cuneus and the parietal lobe in the male group compared with the female group. Previous studies showed that males have significant higher glucose metabolism in bliateral inferior temporal gyrus and frontal lobe, and lower glucose metabolism in patietal lobe, frontal lobe and cingulate gyrus, our results were in line with these findings [12]. Researchers have suggested that cerebellum may play an important role in the pathophysiology of MDD [18, 19]. Wu and Baeken showed that depressive patients with longer depression duration represented significant less metabolic activity in bilateral cerebellum posterior lobe [20], which supported our findings that the metabolic difference existed in cerebellum and the difference was related to depression. Additionally, gender difference in gray matter volumes in hippocampus, orbitofrontal and cingulate cortex were also reported [21–23], which may serve as the structure basis of the gender-related metabolic abnormalities.

To our knowledge, interaction of gender and cancer factors on cerebral metabolic pathology remains unclear. A previous MRI study have shown gender-disease interaction effects in the frontal lobe in MDD patients, which were implicated in the dysfunctional regulation of mood and emotion [24]. Hence, the metabolic abnormality we found in frontal lobe and cuneus may represent disorder of brain metabolic activity based on the pathological function of these regions.

### Correlation analysis with BDI scores

We computed correlation coefficients between the BDI scores and the metabolic intensity in the clusters affected by the gender and cancer factors. The results in Table 4 and Fig. 3 showed a relationship between the brain regions and the mental stress of depressive emotion in parietal lobe, the frontal lobe, the cuneus and the left temporal lobe. The depression-related regions include three clusters with cancer main effect and two clusters with gender main effect. No cluster with gender-by-cancer interaction effect was found significantly related to the mental stress of depressive emotion. In previous studies, researchers focused on the regional metabolism [7], which omitted the voxel-wise information. In this paper, we explored the relationship between voxel metabolic intensity and the mental stress of depressive emotion in cancer patients, and voxels with significant correlation were marked. In addition, the comparison results were corrected by FDR correction (*p* < 0.05, voxel level) instead of FWE correction (*p* < 0.05), as FWE correction may be excessively strict criterium, by which no voxel could survive from the correction.

Similar to the previous research, our results showed that the abnormal metabolism in the parietal lobe and frontal lobe was related to the depression state [7, 25–28]. The results from two-way ANOVA results showed that the metabolism in parietal lobe and frontal lobe was affected by cancer factor. These results suggested that the abnormal metabolism in cancer patients starts at very mild stages in depression [7], which should be paid more attention to as a depressive indicator in clinical nursing care of cancer patients. Regions with gender main effects and abnormal metabolism were found in the cuneus and inferior temporal gyrus, the metabolic intensity of which were related to mental stress of depressive emotion. The previous studies reported that the metabolism in cuneus was positively correlated with severity of mental stress of depressive emotion [27, 28], and the inferior temporal gyrus was related to mental stress of depressive emotion when exposed to negative emotion activation [29]. The abnormal metabolism affected by cancer factor might pose as a risk factor of energy disorder or psychomotor retardation. Which may be the pathological cause of depression; and the gender differences in cerebral metabolism might reflect psychological resilience difference facing the diagnosis of cancer or preventing depression [11, 30–33]. The present work complemented the study of gender differences in cerebral metabolism using the neuroimaging method in cancer patients, and provided support evidence for abnormal cerebral glucose metabolism in cancer patients with mental stress of depressive emotion.

### Methodological limitations

There are some limitations that needs to be addressed in our future work. Firstly, we only used the BDI scale to evaluate the mental status of the cancer patients, but not for the NCs. The mental state (with/without depression) of the NCs was evaluated by psychiatrist. More comprehensive scale tests will performed and provided to fix this problem. Secondly, the sample size in present study is not enough, and varied cancer type may influence the analysis. A replication study needs to be performed in the future on more patients with single type of cancer to examine our findings. Thirdly, some other variables were not included in the present study (such as the coping styles of subjects and hormonal treatments). These limitations may result in a lack of power to demonstrate the relationship between the gender-cancer factors and the mental stress of depressive emotion. In future studies, the gender effects in cancer patients with mental stress of depressive emotion needs to be investigated on more scales and larger sample.

## Conclusions

This article reported that males and females different psychological endurance when facing the diagnosis of cancer or preventing depressive emotion. Moreover, the cerebral abnormal metabolism might be a depressive indicator for cancer patients. These findings may provide objective information to doctors and cancer patients caregivers, so that they can pay more attention to the cancer patients with cerebral abnormal metabolism to improve their life quality. Besides, this work attempt to explore pathophysiological reasons regarding depressive cancer patients in the metabolic way, which may be helpful to diagnose depression, develop effective therapeutic regimen, and also useful for the targeted nursing of cancer patients in clinic.

## Abbreviations

ANOVA: analysis of variant
BDI: Beck Depression Inventory
FDG: fluor-deoxy-glucose
NC: normal control
PET: positron emission tomography
